# The Dietary Inflammatory Index Is Associated with Low Muscle Mass and Low Muscle Function in Older Australians

**DOI:** 10.3390/nu13041166

**Published:** 2021-04-01

**Authors:** Marlene Gojanovic, Kara L. Holloway-Kew, Natalie K. Hyde, Mohammadreza Mohebbi, Nitin Shivappa, James R. Hebert, Adrienne O’Neil, Julie A. Pasco

**Affiliations:** 1 IMPACT Institute for Mental and Physical Health and Clinical Translation, Deakin University, Geelong, Victoria 3220, Australia; k.holloway@deakin.edu.au (K.L.H.-K.); natalie.hyde@deakin.edu.au (N.K.H.); adrienne.oneil@deakin.edu.au (A.O.); julie.pasco@deakin.edu.au (J.A.P.); 2 Biostatistics Unit, Faculty of Health, Deakin University, Geelong, Victoria 3220, Australia; m.mohebbi@deakin.edu.au; 3 Cancer Prevention and Control Program and Department of Epidemiology and Biostatistics, Arnold School of Public Health, University of South Carolina, Columbia, SC 29208, USA; shivappa@email.sc.edu (N.S.); jhebert@mailbox.sc.edu (J.R.H.); 4 Department of Nutrition, Connecting Health Innovations LLC, Columbia, SC 29201, USA; 5 Department of Medicine-Western Health, University of Melbourne, St Albans, Victoria 3021, Australia; 6 University Hospital Geelong, Barwon Health, Geelong, Victoria 3220, Australia

**Keywords:** aged, dietary inflammatory index, dietary patterns, frailty, inflammation, muscle function, muscle mass, sarcopenia

## Abstract

Age-associated chronic, low grade systemic inflammation has been recognised as an important contributing factor in the development of sarcopenia; importantly, diet may regulate this process. This cross-sectional study examined the association of diet-related inflammation with components of sarcopenia. Participants (*n* = 809) aged 60–95 years from the Geelong Osteoporosis Study were studied. Body composition was measured by dual energy X-ray absorptiometry. In this study, low appendicular lean mass (ALM/height^2^, kg/m^2^) was defined as T-score < −1 and low muscle function as Timed-Up-and-Go >10 s over 3 m (TUG > 10). Dietary inflammatory index (DII^®^) scores, based on specific foods and nutrients, were computed using dietary data collected from a food frequency questionnaire. Associations between DII scores and low muscle mass and low muscle function, alone and combined, were determined using linear and logistic regression. After adjusting for covariates, higher DII score was associated with lower ALM/height^2^ (β −0.05, standard error (SE) 0.02, *p* = 0.028), and higher natural log-transformed (ln) (TUG) (β 0.02, standard error 0.01, *p* = 0.035) and higher likelihood for these components combined (odds ratio 1.33, 95% confidence interval 1.05 to 1.69, *p* = 0.015). A pro-inflammatory diet, as indicated by higher DII score, is associated with lower muscle mass, poorer muscle function and increased likelihood for the combination of low muscle mass and low muscle function. Further studies investigating whether anti-inflammatory dietary interventions could reduce the risk of sarcopenia are needed.

## 1. Introduction

Sarcopenia, the loss of muscle mass and function with age, is an important underlying cause of physical disability and frailty, leading to increased risk of falls and fractures, nursing home admission, hospitalisation, decreased quality of life and mortality [1,2,3]. Sarcopenia is common in older adults with an estimated prevalence of 5% to 13% in adults aged 60 to 70 years and 11% to 50% in adults over 80 years of age [4]. In Australia, sarcopenia prevalence has been estimated to be 2.9% for men and 5.9% for women aged 60 to 96 years [5]. The large variability in prevalence is related to the populations studied, different methods used to assess muscle mass, muscle strength and physical performance, and criteria used to define sarcopenia [6,7]. With the ageing of populations, the overall prevalence and number of individuals with sarcopenia is expected to increase. This will present an ever-increasing greater burden on the health care system; making it ever more important to identify novel modifiable risk factors for the prevention and treatment of sarcopenia [8].

Age-associated chronic, low grade systemic inflammation, termed “inflammaging”, has been recognised as an important contributing factor in the development of sarcopenia [9,10,11]. It has been proposed that inflammaging is caused by increased oxidative stress or reduced immune function (immunosenescence) [11]. While the mechanisms are not yet fully understood, there is consensus that inflammaging is accompanied by increased levels of pro-inflammatory cytokines, mainly tumour necrosis factor-alpha (TNF-α) and interleukin-6 (IL-6), and the acute phase protein, C-reactive protein (CRP) [11,12]. More recently, study findings have suggested that inflammaging may stimulate muscle wasting and loss of muscle quality [9,12]. Thus, chronic inflammation may be implicated in the development and progression of sarcopenia.

Importantly, diet may be involved in this process. Specific nutrients, foods and dietary patterns have been associated with biomarkers of inflammation; yet the role of inflammation in the diet as a whole has not been properly investigated [13]. Single nutrient analysis is limited by the high correlation and interactions between many nutrients that make it difficult to distinguish between individual and combined effects [14]. Dietary pattern analysis has emerged as a new, more holistic approach to examine relationships between diet and health outcomes [15]. The dietary inflammatory index (DII^®^) is a validated tool that quantifies the inflammatory potential of nutrients and foods in the context of a dietary pattern [16]. The DII has been used to investigate the association of an inflammatory dietary pattern with various health outcomes associated with ageing, including cardiovascular disease [17], risk of fracture [18], frailty [19,20], and cancer [21]. However, few studies have examined the association of DII scores in relation to sarcopenia and its components [22,23,24,25]. We propose that chronic inflammation is a contributor to sarcopenia and that the inflammatory potential of the diet has a regulatory role on chronic inflammation and thus, sarcopenia.

The overall objective of this study was to examine associations between the inflammatory potential of diet and the components of sarcopenia in men and women aged 60 years and over. Specifically, we aimed to evaluate associations between DII score and (1) lean mass (as a surrogate measure of muscle mass), (2) muscle function and (3) a combination of these two as a representation of sarcopenia.

## 2. Materials and Methods

### 2.1. Study Design

In this population-based study, participants were men and women from the Geelong Osteoporosis Study (GOS). The GOS is an age-stratified sample of men and women aged 20 to 96 years randomly selected from electoral rolls for the Barwon Health Statistical Division in south-eastern Australia. Details of study design, participation and retention have been described elsewhere [26]. The participants were assessed at baseline and have participated in follow-up assessments every few years. Cross-sectional data from two different timepoints, baseline for men (2001–2006) and 15-year for women (2011–2014), were used in this study due to availability of comparable data for the exposure, outcomes and covariates.

The study protocol was approved by the Barwon Health Human Research Ethics Committee. All participants provided written informed consent.

### 2.2. Participants

Individuals aged 60 years and over were included in this analysis, but were excluded if (1) their weight exceeded the limit of the dual energy X-ray absorptiometry (DXA) scanners (≥120 kg), (2) a limb was affected by a prosthesis, plates or screws or had been amputated, (3) a full body scan, and/or a Timed-Up-and-Go (TUG) test was not performed, (4) a food frequency questionnaire (FFQ) was not completed or (5) excessively high or low daily nutritional energy intakes were reported on the FFQ (i.e., <3360 or >16800 kJ/day for men and <2100 or >14700 kJ/day for women) [27].

### 2.3. Outcome Measures

#### 2.3.1. Muscle Mass and Muscle Function

As a surrogate for skeletal muscle mass, lean mass was measured by whole body DXA, which is the preferred method for assessing body composition in a research setting [28]. Appendicular lean mass (ALM, kg) was calculated as the sum of the lean mass measurements for arms and legs, expressed relative to height squared (ALM/height^2^, kg/m^2^).

A Lunar DPX-L (Lunar; Madison, WI, USA) was used to scan the first 544 men at baseline until an upgrade to a GE-Prodigy (Prodigy; GE Lunar, Madison, WI, USA). Cross-calibration was performed on 40 subjects aged 21 to 82 years to ensure comparability of the DXA scanners; no differences were detected in lumbar spine or femoral neck bone mineral density [26]. All scans for the women at 15-year assessment were performed on the GE Lunar Prodigy. The DXA scanner was calibrated three times per week with an anthropometric phantom (Hologic) to preserve the repeatability and accuracy of measures. Muscle function was assessed using a timed “Up-and-Go” (TUG) test, which measures the time taken to rise from a seated position in a chair with no arm rests, walk 3 metres, turn around, walk back and sit down [29].

In this study, a combination of low muscle mass and low muscle function was used as a representation of sarcopenia [28]. Low muscle mass was defined as ALM/height^2^ < 7.87 kg/m^2^ for men and <6.07 kg/m^2^ for women (equal to T-score <−1) [30]. Cut points for ALM/height^2^ were calculated using DXA from a sample of 374 men and 308 women aged 20–39 years from the GOS [30]. As suggested by the European Working Group on Sarcopenia in Older People (EWGSOP), low muscle function can be defined either as low muscle strength or low physical performance [28]. In this study, low muscle function was defined as TUG > 10 s for 3 metres [29]; the TUG is a recognised assessment tool for physical performance [28,31]. Measures of handgrip strength, used to assess low muscle strength, were not available for the recently updated definition of sarcopenia (EWGSOP2) [31].

#### 2.3.2. Exposure: Dietary Inflammatory Index (DII)

Dietary data were collected using the Dietary Questionnaire for Epidemiological Studies (DQES version 2), an FFQ created by Cancer Council Victoria, which was completed by participants at each assessment phase [32]. In this study, the baseline timepoint was used to assess diet from the FFQ for men and the 15-year timepoint for women. The FFQ DQES was designed for use in epidemiological studies and has been validated for the Australian population [33,34]; it captures usual eating habits over the past 12 months covering five types of dietary intake, incorporating 80 items: (1) cereal foods, sweets and snacks, (2) dairy products, meats and fish, (3) fruit, (4) vegetables, and (5) alcoholic beverages on a ten-point frequency scale. Portion sizes are based on dietary data collected on older Australian residents (mean age 61 years), which matches the sample used in our analyses [32]. Analysis of questionnaires for assessment of dietary intakes was undertaken by the Nutritional Assessment Office, Cancer Council Victoria. The output of the FFQ analysis provided estimated intakes of macronutrients and a range of micronutrients which were used to compute DII scores for all participants.

The DII is based upon up to 45 food parameters which have been scored based on reported pro-inflammatory or anti-inflammatory effects on specific inflammatory markers (IL-1β, IL-4, IL-6, IL-10, TNF-α, and CRP) using 1943 peer-reviewed articles published through to December 2010. Details of the development of the DII have been reported elsewhere [16,35] and validation work using inflammatory biomarkers are also available [35,36,37,38,39]. Briefly, the scoring algorithm uses a global reference database (food consumption from eleven populations globally) and food parameter-specific inflammatory effect scores to create an overall DII score for an individual. The DII scores individuals’ diets on a continuum from strongly anti-inflammatory (−8.87) to strongly pro-inflammatory (+7.98).

To calculate DII scores for the participants in this study, dietary intake data were used to calculate an individuals’ intake of food parameters which were then compared to the global reference database. A Z-score for each of the food parameters for each participant was calculated based on the global mean and standard deviation; this was achieved by subtracting the global mean from the amount reported and dividing this value by the standard deviation. The Z-scores were converted to a proportion to minimise the effects of outliers (“right-skewing”). The standardised dietary intake data (proportion) was centred by doubling and subtracting 1 and then multiplied by the inflammatory effect score of each food parameter and summed to obtain an overall DII score for every participant in the study. In this study, a total of 22 of 45 food parameters were available from the FFQ for computing the overall DII scores. These included energy, carbohydrate, protein, total fat, fibre, cholesterol, saturated fat, monounsaturated fat, polyunsaturated fat, omega-3 fatty acids, omega-6 fatty acids, niacin, thiamine, riboflavin, iron, magnesium, zinc, vitamin C, vitamin E, folic acid, beta-carotene and alcohol.

### 2.4. Covariates

Data on age, sex, body fat percentage, height and mobility were collected at all assessment phases. Barefoot standing height (±0.1 cm) was measured using a wall-mounted stadiometer [26]. Measurements of body fat percentage were obtained from whole body DXA scans. Mobility was self-reported and divided into seven categories ranging from “very active” to “bedfast”. For these analyses, two categories of mobility were considered; sedentary (included “sedentary”, “limited”, “inactive”, “chair or bedridden” and “bedfast”) and active (included “very active” and “active”).

### 2.5. Statistical Analyses

All statistical tests were performed using Minitab 17 (Minitab, LLC, State College, PA, USA). The DII was analysed as a continuous variable. Kolmogorov–Smirnov test was used to investigate normality of the data. Independent sample *t* test was used to compare continuous characteristics between sex or other dichotomised factors. If necessary, a non-parametric Mann–Whitney U test was used for this purpose, and a Chi-square test was used for categorical variables. The natural log-transformation was used to normalise TUG scores (used to assess muscle function), which were positively skewed.

Separate linear regression models were used to examine the association between DII and muscle mass and muscle function. A logistic regression model was used to examine the association between DII score and these components combined. Bivariable regression models with no adjustment for participant characteristics were presented (model 1), followed by multivariable regression models that accounted for age (years), sex (male/female) and body fat percentage (%) (model 2). Further adjustments were made for mobility (active/inactive) for ALM/height^2^, and height (m) for ln (TUG). Interaction between co-variables were tested and retained in the final model (model 3) if the interaction term was statistically significant (*p* < 0.05). To test for interaction terms, DII was dichotomised according to the median. Daily nutritional energy intake was not included in the multivariable models as a covariate because energy is already included as a constituent of the DII [36]. Results are presented as standardised beta coefficient (β) and standard error (SE), or as an odds ratio (OR) and 95% confidence interval (95% CI).

## 3. Results

### 3.1. Participants

Out of a total of 2389 individuals (1540 men at baseline and 849 women who participated in the 15-year assessment), 1071 (694 men and 377 women) were ≥60 years. Of these, 262 (163 men and 99 women) were excluded from this analysis because they met one or more of the exclusion criteria: 9 weighed ≥120 kg, 98 were affected by lower limb prostheses, plates or screws, 2 were unilaterally affected by a lower limb amputation, 98 did not provide a full body scan, 112 did not perform a TUG test, 66 did not complete an FFQ, and 20 reported excessively high or low daily FFQ-derived energy intakes. Thus, analyses included data from 809 individuals (531 men and 278 women).

### 3.2. Characteristics of Participants in the Study Sample

Key characteristics are described pooled and by sex in Table 1. Participants’ ages ranged from 60 to 95 years, with 34% identified as female. The DII scores for the sample ranged from −2.7 to 2.5. Median DII scores for women were 0.8 (interquartile range: −0.2 to 1.5) and 0.4 (interquartile range: −0.4 to 1.2) for men. Compared with men, women had higher TUG and DII scores (*p* = 0.02 and *p* = 0.003, respectively), lower ALM/height^2^ and reported lower levels of mobility (*p* < 0.001 and *p* = 0.001, respectively). No differences were detected in proportions of men and women with low muscle mass and low muscle function combined (8.6% vs. 10.9%, *p* = 0.31).

Table 2 shows the total daily energy intake and nutrient intake of participants which was used to calculate DII scores. Calcium intake was similar between men and women; however, men had higher intakes of energy, protein, carbohydrate, fats and alcohol (*p* < 0.001 for all).

### 3.3. Dietary Inflammatory Index and Muscle Mass and Muscle Function

Table 3 shows the results of linear and logistic regression modelling for the association between DII and low muscle mass and low muscle function, alone and combined. A negative association was observed between DII and ALM/height^2^ in the unadjusted model (β = −0.13, SE = 0.04 for model 1). This association persisted after adjustment for age, sex and body fat percentage (β = −0.05, SE = 0.02 for model 2) and for the interaction of age and sex (β = −0.05, SE = 0.02 for model 3). Repeating the statistical analysis with model 2 but including mobility as a covariate did not change the association (β = −0.05, SE = 0.02).

A positive association between DII score and ln (TUG) was observed in the unadjusted model (β = 0.03, SE = 0.01 for model 1). This association remained significant after adjustment for age, sex and body fat percentage (β = 0.02, SE = 0.01 for model 2) and for the interaction of age and sex (β = 0.02, SE = 0.01 for model 3). Repeating the statistical analysis with model 2 but including height as a covariate did not change the association (β = 0.01, SE = 0.01).

Each one-unit increase in DII was positively associated with a 33% increase in combined low ALM/h^2^ plus TUG > 10 s in the unadjusted and adjusted logistic model (OR 1.34, 95% CI 1.08 to 1.67 for model 1; OR 1.33, 95% CI 1.05 to 1.69 for model 2). There were no significant interactions found between covariates.

## 4. Discussion

In this cross-sectional study, higher DII score, indicating a more pro-inflammatory diet, was associated with lower muscle mass, poorer muscle function and higher likelihood for the combination of low muscle mass and low muscle function. The sex*age interaction term identified that the relationship between DII and ALM/height^2^ and ln (TUG) was different between men and women and that the size of this difference increased with increasing age.

In this study, higher DII score (indicating a more pro-inflammatory diet) was associated with lower ALM/height^2^, indicating lower muscle mass. Other studies examining the relationship between DII and muscle mass have reported similar results. In a prospective longitudinal study of 1098 individuals aged 50 to 79 years from the Tasmanian Older Adult Cohort Study (TASOAC), inverse associations were shown between energy-adjusted DII scores and appendicular lean mass in men but not in women after controlling for age and percent body fat (semi-adjusted model) [24]. Findings from a study of 466 Chinese boys and girls aged 6 to 9 years reported that DII score was inversely associated with relative appendicular skeletal muscle mass (ASM/height^2^) [40]. In a longitudinal study with 494 female participants aged 21 to 89 years from the GOS, while the DII was not predictive of skeletal muscle index (ALM/height^2^) significance increased with adjustment; thus, suggesting a higher DII score was associated with increases in skeletal muscle index [23]. Together, these findings highlight the potential role for overall diet quality based on the inflammatory potential of diet in the maintenance of skeletal muscle mass across the life course.

Other studies that have looked at anti-inflammatory dietary patterns like the Mediterranean diet and muscle mass have produced differing results [41,42]. In a cross-sectional study of women aged 18 to 79 years from the Twins UK study, higher adherence to a Mediterranean diet was associated with higher FFM% (fat-free mass/weight × 100) after adjustment for age, physical activity, smoking, energy and protein intake and misreporting; specifically, FFM% was 1.0% higher in the highest quartile (Q4) compared to the lowest quartile (Q1) [41]. In contrast, in a study conducted in Iran among community-dwelling men and women with an average age of 66 years, no differences in mean muscle mass were detected in the higher tertiles of a Mediterranean dietary pattern compared with the lower tertiles; although the direction of the association was as expected (i.e., lower adherence to a Mediterranean dietary pattern was associated with lower muscle mass) [42]. These inconsistencies may be due to a range of factors including insufficient sample size, the use of samples with different age ranges (e.g., some including both pre and postmenopausal women), different ranges of the DII scores and the different settings.

Another finding of our study was that higher DII score is associated with higher ln (TUG). Handgrip strength, a clinical marker of poor mobility, and gait speed can also be used to assess low muscle function for the diagnosis of sarcopenia [28]. Several studies have explored these measures, but results have been inconsistent. In a cohort study of 1948 individuals aged 60 years or older from the Seniors-ENRICA study, higher DII score was associated with slow gait speed, as a low score in the Short Physical Performance Battery (SPPB) test [20], which is somewhat comparable to our study findings. In a study of 321 individuals aged 70 to 85 years, low gait speed and low grip strength were positively associated with higher DII scores [43]. Furthermore, in a cross-sectional study of 78 frail individuals aged 65 years or older from South Korea, a higher SPPB score was associated with lower levels of TNF-α, suggesting that improving muscle function may lower levels of inflammation [44]. Conversely, no significant associations have been observed between DII and gait speed or handgrip strength in other studies [22,24,40]. The inconsistency of results could be due to different methods used to assess muscle function, age-group differences and limited DII score ranges. More research is therefore required to determine the effects of dietary inflammation on muscle function in older adults.

The final component considered in this study was a combination of low muscle mass and low muscle function as a representation of sarcopenia. We found that higher DII score was associated with a higher likelihood for these components combined. Our findings are in agreement with a cross-sectional study of 300 individuals aged 55 years or older from Iran by Bagheri et al. [22], who found that those in the top tertile of DII had higher odds of sarcopenia than those in the bottom tertile. In a study of 1344 postmenopausal Korean women aged 50 years or older, a pro-inflammatory diet, as determined by DII score over the median, was associated with increased odds for sarcopenic obesity. However, this result was attenuated and did not reach statistical significance after adjustment for age, family income, regular exercise, education status, smoking and female hormone supplements [25]. Interestingly, a pro-inflammatory diet was associated with increased odds for osteosarcopenic obesity in the adjusted model [25]. However, a direct comparison between these results and ours is made difficult by several factors; sarcopenic obesity is a distinct condition [31], two different criteria were used to define sarcopenia (low muscle mass and function vs. low muscle mass alone) and muscle mass was adjusted for body size in different ways (ALM/height^2^ vs. ASM/weight %). Cut-off values also differ because of ethnicity, body size, lifestyles and culture between European and Asian populations [45], and there is no consensus about which method is best for adjusting for body size [31].

To date, evidence that a pro-inflammatory diet is associated with sarcopenia has been limited. Previous studies have mainly focused on the association of “healthy eating”, high fruit and vegetable intake, and Mediterranean anti-inflammatory dietary patterns with sarcopenia [42,46,47,48,49]. Our findings support those observed by Hashemi et al. [42] who found that a Mediterranean dietary pattern was associated with lower odds for EWGSOP-defined sarcopenia among community-dwelling men and women with an average age of 66 years. Given that the inflammatory potential of the Mediterranean diet is comparable to a DII score of −3.96, indicating a strong anti-inflammatory potential, in a similar way, these results are consistent with our study findings [50]. In contrast, Chan, Leung and Woo [47] found no association between Mediterranean Diet Score (MDS) and the Asian Working Group for Sarcopenia (AWGS)-defined sarcopenia in a prospective cohort study of community-dwelling Chinese men and women aged 65 years and older. The absence of associations may be due to the differences in the Chinese diet compared to the traditional Mediterranean diet. Additionally, cut points for muscle mass were lower (<7.0 kg/m^2^ for men and <5.4 kg/m^2^ for women) than those used in this study, which may have affected the case ascertainment of sarcopenia.

Consistent with the findings of this study, other studies have suggested that a pro-inflammatory diet, as measured by the DII, is associated with increased hip fracture risk and frailty, which are associated with loss of muscle mass and/or function [18,19,51,52]. Research indicates that chronic low-grade inflammation plays a role in the development of sarcopenia, and that diet plays a role in the regulation of chronic inflammation, supporting the findings of this study that the inflammatory potential of the diet may be a modifiable risk factor for sarcopenia [9,13,53].

There were several strengths to this study. The secondary analysis of existing data from the GOS allowed for access to a large data set. Not only was this efficient but the random sampling method used in the GOS strengthened the external validity of this study by achieving a sample that was representative of the underlying population [26]. Objective measures were used to assess muscle mass and muscle function. Furthermore, a systematic approach was adopted for addressing confounding and effect modification with adjustment for a number of variables. The validity of the Cancer Council Victoria FFQ has been assessed against weighed food records in Australian men and women ranging from 31 to 75 years [33] and in young to middle-aged women [34] with good agreement; thus, confirming that the FFQ used was a valid tool in the assessment of dietary intake in our study sample of Australian men and women.

Despite its strengths, our study had several limitations. The primary limitation of cross-sectional studies is the inability to account for temporality, and as a result, causality cannot be established. Reliance on long-term memory for some self-reported data may have affected the accuracy of dietary and lifestyle self-reported data, resulting in recall bias and increased random measurements error [54,55]. Despite using objective measures to confirm some self-reported data, biases may still exist. As well, the presence of selection bias due to non-response and attrition rates cannot be excluded. Additionally, the fact that data were pooled from different study periods for men and women may have introduced bias. Data also may have been affected by the exclusion criteria; as a consequence, the study findings may not be applicable to individuals who weigh ≥120 kg or who are affected by lower limb prostheses, plates or screws. The original definition of sarcopenia by EWGSOP focussed on the detection of low muscle mass. More recent definitions have turned attention to low muscle strength as the primary diagnostic criterion of sarcopenia [31,56]. In the absence of muscle strength measures in this data set, we have not adopted the latest version of the definition. Furthermore, the absence of data on 23 parameters may have limited the range of DII scores, which appear to be somewhat narrower than other studies [57]. This may have contributed to the narrow effective range of the DII score, which is about half of that normally observed in other studies that typically range from about −5 to +5 [57]. Increasing the effective range of the independent variable often increases magnitude of the observed effect [58]. Therefore, our results actually may underestimate the relationship between DII score and the combined low muscle mass and low muscle function components.

## 5. Conclusions

A pro-inflammatory diet, as indicated by higher DII score, is associated with lower muscle mass, poorer muscle function and increased likelihood for the combination of low muscle mass and low muscle function among older Australian men and women. These results support the notion that a pro-inflammatory diet negatively affects muscle mass and muscle function and exacerbates the risk of developing sarcopenia. Future studies could consider the relationship between DII and the sarcopenia trajectory and investigate whether anti-inflammatory dietary interventions could reduce the risk of sarcopenia.

## Figures and Tables

**Table 1:** Key Characteristics of the Participants; Data are Shown for All, and According to Sex.

Characteristics	Total (*n* = 809)	Females (*n* = 278)	Males (*n* = 531)	*p* Value
DII score	0.6 (−0.3, 1.3)	0.8 (−0.2, 1.5)	0.4 (−0.4, 1.2)	0.003
Age (yr)	66.4 (72.4, 78.8)	70.6 (65.0, 75.3)	74.0 (67.0, 81.3)	<0.001
Height (cm)	167.9 ± 8.7	159.9 ± 6.0	172.1 ± 6.6	<0.001
Weight (kg)	78.2 ± 13.7	73.5 ± 14.6	80.6 ± 12.5	<0.001
BMI (kg/m^2^)	27.7 ± 4.5	28.8 ± 5.6	27.2 ± 3.8	<0.001
Body fat (%)	32.0 ± 10.1	42.1 ± 8.0	26.7 ± 6.4	<0.001
ALM/h^2^ (kg/m^2^)	7.7 ± 1.1	6.6 ± 0.8	8.2 ± 0.9	<0.001
TUG (s)	8.9 (7.6, 10.3)	9.1 (7.8, 10.8)	8.6 (7.6, 10.1)	0.02
Mobility level (active) *	533 (66.2)	161 (58.8)	372 (70.0)	0.001
ALM/h^2^ cutpoint (below) ^†^	257 (31.8)	74 (26.6)	183 (34.5)	0.02
TUG >10 s (yes)	183 (22.6)	76 (27.3)	107 (20.1)	0.02
Low ALM/h^2^ and TUG > 10 s (yes)	82 (10.1)	24 (8.6)	58 (10.9)	0.31

DII, dietary inflammatory index; ALM/h^2^, appendicular lean mass/height^2^; TUG, Timed-Up-and-Go. Data are presented as mean ± standard deviation, median (interquartile range) or *n* (%). Comparison of characteristics between male and female participants was performed using independent sample *t* test with parametric continuous variables, Mann–Whitney U test with non-parametric continuous variables, and Chi-square test with categorical variables. * Missing values: 4 for mobility level. ^†^ ALM/height^2^ cutpoints: <7.87 kg/m^2^ for men, <6.07 kg/m^2^ for women.

**Table 2:** Total Daily Energy and Nutrient Intake of Participants; Data is Shown for All, and According to Sex.

Nutrient	Total (*n* = 809)		Females (*n* = 278)		Males (*n* = 531)		*p* Value
	Median	IQR	Median	IQR	Median	IQR	
Energy (kJ)	6927.9	(5593.3, 8632.0)	5991.5	(4784.0, 7438.5)	7353.2	(6195.3, 9230.9)	<0.001
Protein (g)	78.2	(63.0, 96.4)	70.0	(55.1, 88.5)	83.1	(67.6, 100.0)	<0.001
Carbohydrate (g)	186.7	(146.7, 234.6)	154.0	(119.8, 194.2)	205.0	(164.0, 251.1)	<0.001
Total fat (g)	66.8	(51.0, 85.4)	57.5	(45.4, 72.5)	71.3	(56.4, 92.3)	<0.001
Saturated fats (g)	26.4	(19.6, 34.6)	23.4	(17.7, 30.5)	28.1	(21.0, 36.4)	<0.001
Polyunsaturated fatty acids (g)	10.6	(7.2, 14.9)	8.7	(6.1, 12.1)	12.1	(8.3, 16.2)	<0.001
Monounsaturated fatty acids (g)	23.2	(17.6, 30.1)	20.1	(15.4, 25.5)	24.6	(19.2, 32.0)	<0.001
Sugar (g)	86.4	(67.3, 111.8)	74.1	(55.1, 95.4)	93.3	(74.0, 120.8)	<0.001
Starch (g)	97.4	(73.0,126.2)	78.8	(60.2, 99.8)	109.3	(85.0, 138.6)	<0.001
Fibre (g)	20.5	(16.4, 26.3)	18.8	(14.6, 23.4)	21.5	(16.9, 27.8)	<0.001
Alcohol (g)	5.6	(0.3, 21.9)	2.1	(0.0, 12.7)	10.1	(0.9, 26.7)	<0.001
Beta-carotene (μg)	2358.2	(1680.8, 3323.5)	2246.8	(1624.0, 3089.6)	2440.9	(1710.6, 3539.8)	0.02
Calcium (mg)	849.0	(672.3, 1061.7)	848.8	(648.6, 1113.3)	849.1	(683.0, 1047.1)	0.67
Cholesterol (mg)	239.2	(182.0, 316.3)	221.8	(167.5, 297.9)	249.6	(190.1, 322.0)	0.001
Folate (μg)	255.0	(197.9, 321.1)	227.3	(185.7, 291.7)	271.2	(214.8, 335.6)	<0.001
Iron (mg)	11.7	(9.0, 14.7)	10.3	(7.7, 13.3)	12.4	(9.6, 16.0)	<0.001
Magnesium (mg)	270.2	(217.3, 336.3)	247.8	(197.0, 310.4)	282.6	(227.6, 346.2)	<0.001
Niacin (mg)	33.7	(26.7, 42.1)	30.4	(22.8, 37.9)	35.9	(28.5, 44.4)	<0.001
Phosphorus (mg)	1393.7	(1100.6, 1708.0)	1281.7	(1025.5, 1616.7)	1440.1	(1150.8, 1732.3)	0.001
Potassium (mg)	2684.6	(2196.3, 3270.8)	2451.4	(1979.5, 3015.7)	2830.4	(2300.8, 3379.9)	<0.001
Retinol (μg)	965.8	(609.7, 966.7)	686.7	(532.0, 858.4)	810.4	(641.3, 1020.1)	<0.001
Riboflavin (mg)	2.1	(1.7, 2.7)	2.0	(1.6, 2.5)	2.2	(1.7, 2.8)	0.002
Sodium (mg)	2163.1	(1688.1, 2767.4)	1840.5	(1459.1, 2306.2)	2317.1	(1875.9, 2976.4)	<0.001
Thiamine (mg)	1.4	(1.1, 1.8)	1.2	(1.0, 1.5)	1.5	(1.2, 2.0)	<0.001
Vitamin C (mg)	102.1	(71.6, 148.5)	92.1	(67.8, 125.7)	107.0	(75.7, 160.6)	0.001
Vitamin E (mg)	5.9	(4.6, 7.6)	5.4	(4.0, 7.0)	6.2	(4.8, 8.0)	<0.001
Zinc (mg)	10.2	(8.1, 12.8)	9.1	(7.1, 11.6)	10.7	(8.8, 13.1)	<0.001

IQR, interquartile range. Comparison of daily dietary intakes between females and males was performed using Mann–Whitney U test.

**Table 3:** Linear and Logistic Regression Results for the Association between DII Score and Low Muscle Mass and Low Muscle Function, Alone and Combined, for All Participants, Geelong Osteoporosis Study (GOS), 2001 to 2014.

Outcome Variable	Model 1 *			Model 2 ^†^			Model 3 ^‡^		
	β	SE	*p* Value	β	SE	*p* Value	β	SE	*p* Value
ALM/h^2^ (kg/m^2^)	−0.13	0.04	<0.001	−0.05	0.02	0.036	−0.05	0.02	0.028
ln(TUG) (s)	0.03	0.01	<0.001	0.02	0.01	0.028	0.02	0.01	0.035
	**OR**	**95% CI**	***p*** ** Value**	**OR**	**95% CI**	***p*** ** Value**			
Low ALM/h^2^ and TUG > 10 s (yes)	1.34	1.08, 1.67	0.007	1.33	1.05, 1.69	0.015			

β, standardised beta coefficient; SE, standard error; ALM/h^2^, appendicular lean mass/height^2^; ln(TUG), natural log-transformed Timed-Up-and-Go; OR, odds ratio; CI, confidence interval. Standardised beta coefficients and standard errors and odds ratios and confidence intervals are for DII scores. * Model 1: unadjusted. ^†^ Model 2: adjusted for age, sex and body fat percentage. ^‡^ Model 3: adjusted for co-variables in model 2 as well as sex*age interaction term.

## Data Availability

The data that support the findings of this study are available from the corresponding author upon reasonable request.
